# Complete Genome Sequence of a Variant *Pyrrhula pyrrhula polyomavirus 1* Strain Isolated from White-Headed Munia (*Lonchura maja*)

**DOI:** 10.1128/genomeA.01172-16

**Published:** 2016-12-01

**Authors:** Szilvia Marton, Károly Erdélyi, Ádám Dán, Krisztián Bányai, Enikő Fehér

**Affiliations:** a Institute for Veterinary Medical Research, Centre for Agricultural Research, Hungarian Academy of Sciences, Budapest, Hungary; b Veterinary Diagnostic Directorate, National Food Chain Safety Office, Budapest, Hungary

## Abstract

A novel variant of finch polyomavirus has been identified and sequenced from a diseased white-headed munia (*Lonchura maja*).

## GENOME ANNOUNCEMENT

Polyomaviruses of birds have 4,981- to 5,421-bp-long double-stranded circular DNA genomes that encode the small and large tumor antigens, the VP1, VP2, and VP3 structural proteins, and the putative VP4 or ORF-X protein (1–4). At present, seven avian polyomavirus species have been classified into the *Gammapolyomavirus* genus of the *Polyomaviridae* family, including the *Aves polyomavirus 1, Anser anser polyomavirus 1*, *Pyrrhula pyrrhula polyomavirus 1*, *Corvus monedula polyomavirus 1*, *Serinus canaria polyomavirus 1*, *Cracticus torquatus polyomavirus 1*, and *Pygoscelis adeliae polyomavirus 1* (1). Avian polyomaviruses may cause fatal infection with severe organ failure and developmental disorders, particularly in young hosts (2–5).

In this study, we report the complete genome sequence of a polyomavirus strain detected in white-headed munia (*Lonchura maja*) in a flock of a Hungarian aviculturist. Histopathological examination revealed liver failure, nephritis, and myocarditis in the affected bird. From the pooled liver and kidney sample, polyomavirus DNA was detected by a broad-spectrum nested-PCR assay (6) coupled with Sanger sequencing of the amplicon. The whole viral genome was amplified with newly designed inverse primers. The purified PCR products were used for library preparation with the NEBNext Fast DNA Fragmentation & Library Prep Set for Ion Torrent (New England BioLabs) and the Ion Xpress Barcode Adapters (Life Technologies). The emulsion PCR and templated bead enrichment was carried out with OneTouch 2 Instrument and the Ion OneTouch ES (Life Technologies). Sequencing was performed on an Ion Personal Genome Machine (Life Technologies). Sequences were trimmed, assembled, and aligned with CLC Genomics Workbench software version 7 (CLC bio) (7).

The genome of the Hungarian polyomavirus strain, 14534/2011, was 5,284 bp in length and showed the highest overall nucleotide sequence homology of 91.1 and 91.2% with *Pyrrhula pyrrhula polyomavirus 1* sequences identified in Eurasian bullfinch (*Pyrrhula pyrrhula griseiventris*; accession no. DQ192571) and Gouldian finch (strain 1-2012 from *Erythrura gouldiae*; accession no. KC660158), respectively (2). The genomic sequence of the finch origin polyomavirus strain 1209 (accession no. KT302407) was only distantly related to these strains (60.0 to 60.2% similarity) (8). The sizes and locations of predicted open reading frames (ORFs) of the newly determined polyomavirus genome were correspondent with the genomes of closely related strains. In addition, the nucleotide and amino acid sequence similarity values for the large T antigen were up to 91.4% and 96.4%, respectively. Thus, taking into account the most recent species demarcation criterion of polyomaviruses, which defines a new polyomavirus species if the genetic distance for the large T-antigen-coding genomic region is >15%, the Hungarian finch polyomavirus could be unequivocally classified into the common gammapolyomavirus species *Pyrrhula pyrrhula polyomavirus 1* (1).

### Accession number(s).

The whole genome sequence of the strain 14534/2011 has been deposited in GenBank under the accession no. KX756154.
